# Fahr syndrome discovered in adulthood revealing a rare *GNAS* mutation in pseudohypoparathyroidism type 1a in a Tunisian family

**DOI:** 10.1002/ccr3.5849

**Published:** 2022-05-16

**Authors:** Wided Debbabi, Dayssem Khelifi, Issam Kharrat, Slim Samet

**Affiliations:** ^1^ Department of Endocrinology Faculty of Medicine of Sousse Ibn Jazzar University Hospital Kairouan University of Medicine Kairouan Tunisia

**Keywords:** Albright's hereditary osteodystrophy, Fahr syndrome, GNAS mutation, molecular analysis, pseudohypoparathyroidism

## Abstract

Pseudohypoparathyroidism (PHP) indicates a rare heterogeneous group of disorders characterized by hypocalcemia, hyperphosphatemia, increased serum concentration of parathyroid hormone (PTH), and insensitivity to the biologic activity of PTH. One of its most common types is PHP‐1a. In this report, we present a familial PHP‐1a and a novel mutation of the *GNAS* gene.

## Abstract 2

PHP1a is one of the rare and little‐known pathologies that can take a long time to be discovered. It associates a particular morphotype, subcutaneous calcifications and bone and renal resistance to parathormone. It is an inherited condition, which results from an abnormality in the GNAS gene. It is usually diagnosed in childhood. and few studies have addressed its natural history. Our report illustrates the natural history of a patient with PHP1A, which went undiagnosed until the age of 31 years. Through this clinical case, we emphasize the importance of a complete phosphocalcic balance in order to find and recognize the typical biochemical alterations associated with this pathology (hypocalcemia, hyperphosphatemia and high levels of PTH), the need for a detailed clinical examination because the phenotypic appearance often escapes notice and the identification of the responsible mutation in the index case is useful for screening other family members avoiding late diagnosis.

## 1 INTRODUCTION

Fahr syndrome is defined by the presence of calcifications of the basal ganglia bilateral and symmetrical, particularly affecting patients with dysparathyroidism, more rarely pseudohypoparathyroidism (PHP).^1^

The disease was first described and named by Fuller Albright and colleagues in 1942.^2^ It is a heterogeneous group of disorders characterized by hypocalcemia, hyperphosphatemia, increased serum concentration of parathyroid hormone (PTH), and insensitivity to the biologic activity of PTH.^2^ Several variants of PHP have been identified. The molecular defects in the GNAS gene encoding the alpha subunit of the stimulatory G protein (Gs*α*) contribute to at least 3 different forms of the disease: PHP type 1a, PHP type 1b, and pseudopseudohypoparathyroidism.^3^

PHP‐1 is further divided into three different subtypes: 1a, 1b, and 1c.^3^ PHP‐1c, which is identical to PHP‐1a in terms of the presence of AHO and hormone resistance, but in contrast to PHP‐1a, in vitro assessment of Gs‐alpha protein activity, reveals no abnormality, and mutations of GNAS are usually not observed.^4^

The exact prevalence of PHP is unknown^5^ because the investigators did not confirm the clinical diagnosis of PHP by a molecular analysis for most of the patients.

Herein, we report a case of Fahr syndrome which allowed the diagnosis of very rare *GNAS* mutation in familial pseudohypoparathyroidism type 1a.

## 2 CASE PRESENTATION

A 31‐year‐old man was born at full term. There was a distant familial consanguinity. Followed since the age of 15 for recurrent convulsive seizures (initial etiological investigation was negative specifically the serum calcium level was strictly normal), put on anti‐epileptic treatment. Recently, the patient has reported memory disorders, a brain *magnetic resonance imaging* (MRI) has been carried out and revealed the presence of basal ganglia calcification. The patient was referred to our department for further explorations.

At admission, he was conscious with Glasgow Coma Scale of 15 points, afebrile, and had stable vital signs. He had positive Chvostek and Trousseau signs without tetany. The neurologic examination revealed no focal signs.

**FIGURE 1:**
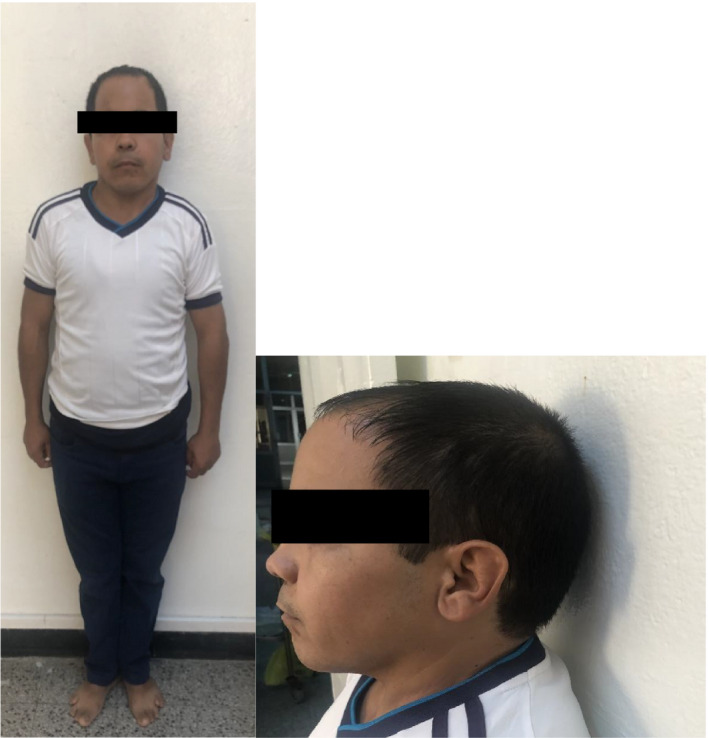
Photographs of our index case: short stature, short neck, mandibular propulsion

**FIGURE 2:**
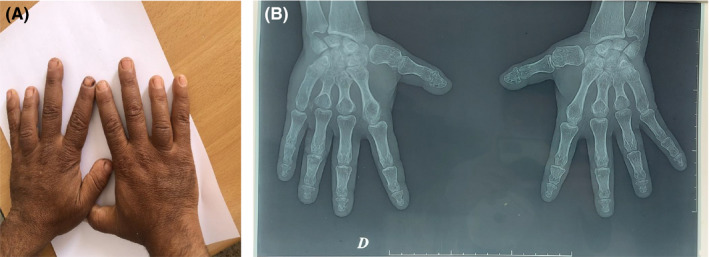
Brachydactyly. (A) Brachydactyly of the hands. Shortened fourth digits in both hands are very remarkable. (B) Radiograph of both hands: brachydactyly due to shortening of metacarpals

**FIGURE 3:**
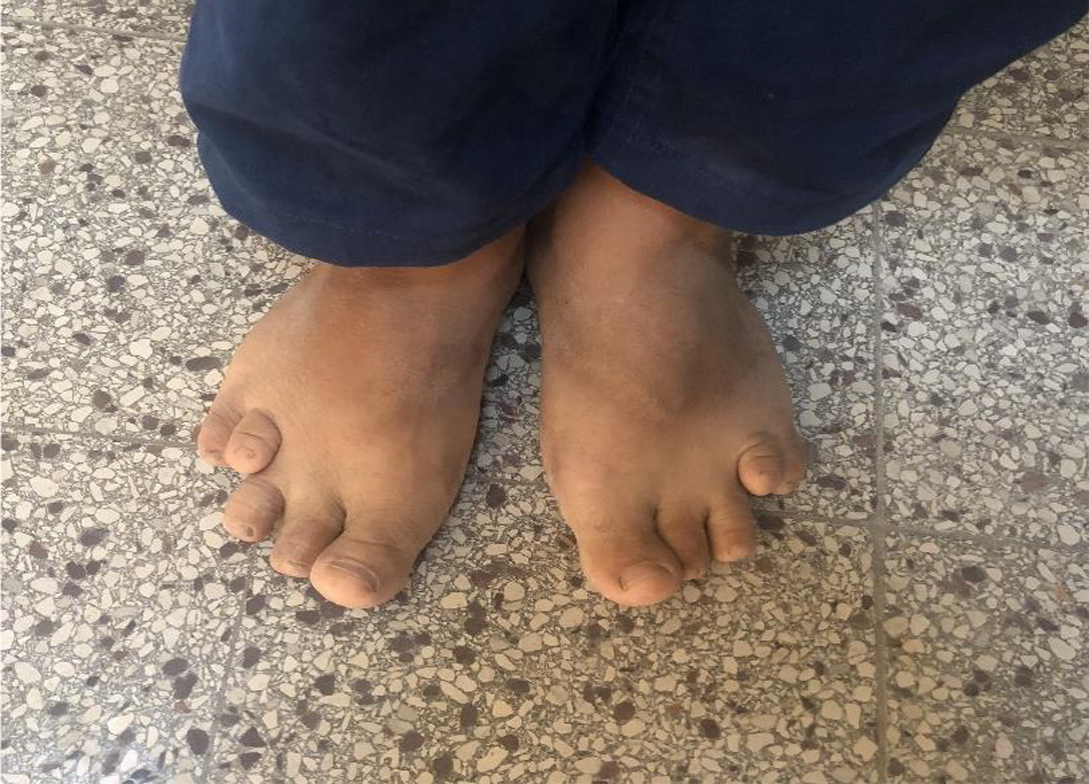
Shortening of all toes (particularly the fourth)

Clinical examination showed short stature (height 1.39 m <5^th^ percentile), weight of 43 kg (<5^th^ percentile) with body mass index of 22.7 kg/m^2^, round face, dental hypoplasia, diastema, short neck, brachymetacarpy, brachymetatarsia, and subcutaneous ectopic ossifications (Figures 1, 2 and 3). Electrocardiogram revealed a prolonged QTc interval (0.47 sec). The ophthalmologic examination showed a bilateral subcapsular cataract.

Laboratory findings showed severe hypocalcemia, with albumin correction, 1.15 mmol/L (normal range 2.2–2.6 mmol/L), hyperphosphatemia 2.76 mmol/L (normal range 0.87–1.45 mmol/L), while the serum PTH level was 320 pg/ml (normal range 15–65 pg/ml) with low calcium in the 24‐h urine collection 26.6 mg/24 h (normal range 100–300 mg/24 h), normal renal function, normal vitamin D (28 ng/ml, normal range 20–40 ng/ml), and normal serum magnesium 0.82 mmol/L (normal range 0.7–1.05 mmol/L).

Thyroid function showed elevated thyroid‐stimulating hormone (TSH) 8.62 uIU/ml (normal range 0.27–4.2 uIU/ml), with normal free thyroxine of 16.51 pmol/L (normal range 12–22 pmol/L). He was negative for anti‐thyroid peroxidase antibody (TPOA), and thyroid ultrasonography was normal. Basal growth hormone (GH) was <0.03 ng/ml which could be probably explained by the resistance to the action of GHRH (GHRH was not performed). Cortisol, follicle‐stimulating hormone (FSH), and serum testosterone levels were within the normal range.

Depending on the history, clinical manifestations, and initial blood investigation, the diagnosis of PHP type 1a was considered. After explaining to the patient, the implications of the diagnosis, we were able to obtain consent to further investigation.

The family investigation revealed the presence of the same clinical and biological presentation in his sister and his younger brother and the discovery of asymptomatic hypocalcemia in the mother. His father was healthy and normal (Table 1).

**TABLE 1:** Laboratory findings

	Index case	Brother	Sister	Mother	Father	Normal range
Calcium (mmol/L)	1.15	1.78	1.98	2	2.48	2.2–2.6
Phosphatemia (mmol/L)	2.76	1.94	1.62	1.74	0.92	0.87–1.45
Parathyroid Hormone (pg/ml)	320	467	280	442	54	15–65
Magnesium(mmol/L)	0.82	0.81	0.86	0.81	0.87	0.7–1.05
Creatinine (μmol/L)	64	76.9	62	70	72	55–115
25‐OH‐Vitamin D (ng/ml)	28	30	27	26	29	20–40
TSH (µUI/ml)	8.62	6.68	7.24	4.1	‐	0.27–4.2
FT4 (pmol/L)	16.82	14.22	15.61	17.26	‐	12–22
GH (ng/ml)	<0.03	<0.03	<0.03	4.2	‐	0.09–6.29
FSH (mIU/ml)	4.63	9.26	8.2	‐	‐	2–15
Testosterone(ng/ml)	5.2	8.12	‐	‐	‐	2.8–8
Estradiol(pg/ml)	‐	‐	54.06	‐	‐	12.5–166

A mutation analysis on the *GNAS* gene was performed for the patient and his family (parents, brother, and sister). Genomic DNA was isolated from peripheral blood. *GNAS* exons and intron/exon boundaries were sequenced (reference sequence NM_000516) with identification of the very rare frameshift mutation c.860_861del p (Val287 Aspfs*12) in heterozygosity, in the location 20q13.2‐q13.32 exon 11 of *GNAS* gene which is absent from international databases (GnomAD). This mutation is detected in our index case, in his mother, sister, and brother. This variation was inherited from his mother. The diagnosis of PHP‐1a with AHO and PTH, TSH, and probably GHRH resistance was retained.

The patient began intravenous infusion with ten percent calcium gluconate and 0.5 *μ*g/day of 1*α*(OH)D_3_. The clinical conditions rapidly improved, and the values of calcium gradually increased to allow, on the third day of hospitalization, a transition to oral supplementation. We started treatment with 3 g/day of calcium lactate hydrate and 1 *μ*g/day of 1*α*(OH)D_3_. During follow‐ups, his calcemia levels were normal and PTH reduced to 98 pg/ml. He had no further seizures. Simultaneous therapeutic management of other members of his family was carried out.

## 3 DISCUSSION

We report a case of Fahr syndrome discovered in adulthood at the age of 31 during memory disorders and hypocalcemic convulsions progressing from childhood which allowed the diagnosis of familial PHP type 1a.

The diagnosis of PHP‐1a was retained in accordance with the recommendations of the recently published first consensus statement on the diagnosis and management of PHP‐related disorders. The diagnosis should be based on clinical and biochemical characteristics, which will vary according to the age of the patient. The diagnosis of AHO should be based on the presence of major criteria (brachydactyly due to premature fusion of the epiphyses and short stature by adulthood) and additional criteria (stocky build, round facies, and ectopic ossifications). Obesity, dental manifestations, and cognitive impairment are present in a subgroup of patients.^6^

In our case, the principal manifestation was seizure and memory disorders resulted from Fahr syndrome which is a rare neurodegenerative disease characterized by bilateral basal ganglia calcification. Tetany and epilepsy were reported to be the most common symptoms in PHP patients. The prevalence of epilepsy was 47.1, and 94.6% for intracranial calcification with a positive correlation with seizures in a Chinese cohort of PHP.^7^ It was common in PHP patients, nevertheless the symptoms of the former disease usually occur between the fourth and sixth decade of life.^8^

In patients with PHP‐1a, resistance to PTH is usually absent at birth and evolves over time (from the neonatal period to 22 years); the first biochemical abnormalities to appear are increased serum PTH and phosphorous levels, whereas hypocalcemia develops gradually 4–5 years later^9^ which explains the normality of serum calcium level during the initial etiological investigation of our patient at the age of 15 years.

Other associated endocrine features that support the diagnosis are as follows: early‐onset hypothyroidism due to TSH resistance, which is the most common associated endocrine alteration, being present in nearly 100% of PHP‐1a patients; hypogonadism (due to FSH and LH resistance); and GH deficiency (due to GHRH resistance).^10^ In our case, PTH, TSH, and probably GHRH resistance were found at the same time, which induced electrolytes disturbance, hypothyroidism. GH deficiency partially explains the final height deficit in patients with PHP‐1a, with mean heights of 155 cm in men.^11^ In our case, the final height of the patient was 139 cm.

Genetically, PHP type 1a is due to a heterozygous loss of function of the alpha subunit of a G protein (Gs*α*), due to a GNAS mutation on the maternal allele of the chromosome 20q13.3, with autosomal dominant inheritance.^12^ This intracellular protein is responsible for the production of cyclic AMP (cAMP) in response to PTH, and the reduced G protein activity is the molecular basis for hormone resistance in this disorder.^7^ The phenotype is most likely explained by the fact that some tissues (thyroid, pituitary, renal proximal tubules, and gonads) express Gs*α* predominantly from the maternal allele, while the paternal is silenced through yet unknown mechanisms.^13^ In this case, a novel heterozygous frameshift mutation, NM_000516 (*GNAS*): c.860_861del p.(Val287Aspfs*12), in exon 11 of the *GNAS* gene was identified for PHP‐1a diagnosis. This very rare variant (absent GnomAD) was also detected in the brother, the sister, and the mother, thus confirming its maternal transmission in the autosomal dominant mode.

## 4 CONCLUSION

Our case is about a PHP‐1a with a very rare frameshift mutation in *GNAS* gene in a patient presenting with characteristics of AHO, as well as TSH and GH resistance. There are over 340 reported GNAS mutations leading to PHP type 1a, and the identification of the responsible mutation in the index case is useful for screening other family members avoiding late diagnosis. The mutation in our case has not been reported in literature and adds to the spectrum of genetic mutations related to PHP.

## AUTHOR CONTRIBUTION

Dayssem Khelifi conceived, analyzed, and drafted the manuscript. Wided Debbabi contributed to drafting and critical revision of the manuscript. Issam Kharrat and Slim Samet analyzed the case report.

## CONFLICTS OF INTEREST

The authors declare no conflicts of interest.

## ETHICAL APPROVAL

Ethical approval for this case report was not required.

## CONSENT

Written informed consent for publication of this case report was obtained from the patient.

## Data Availability

No data were available.
